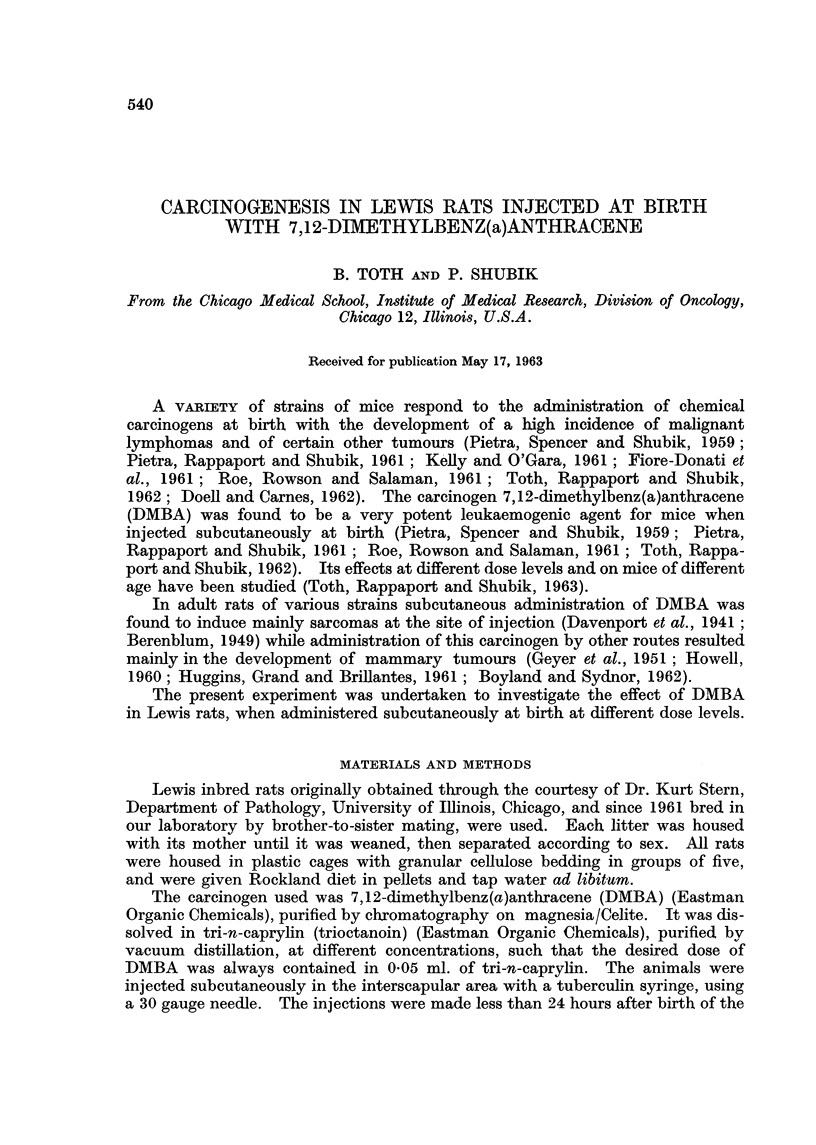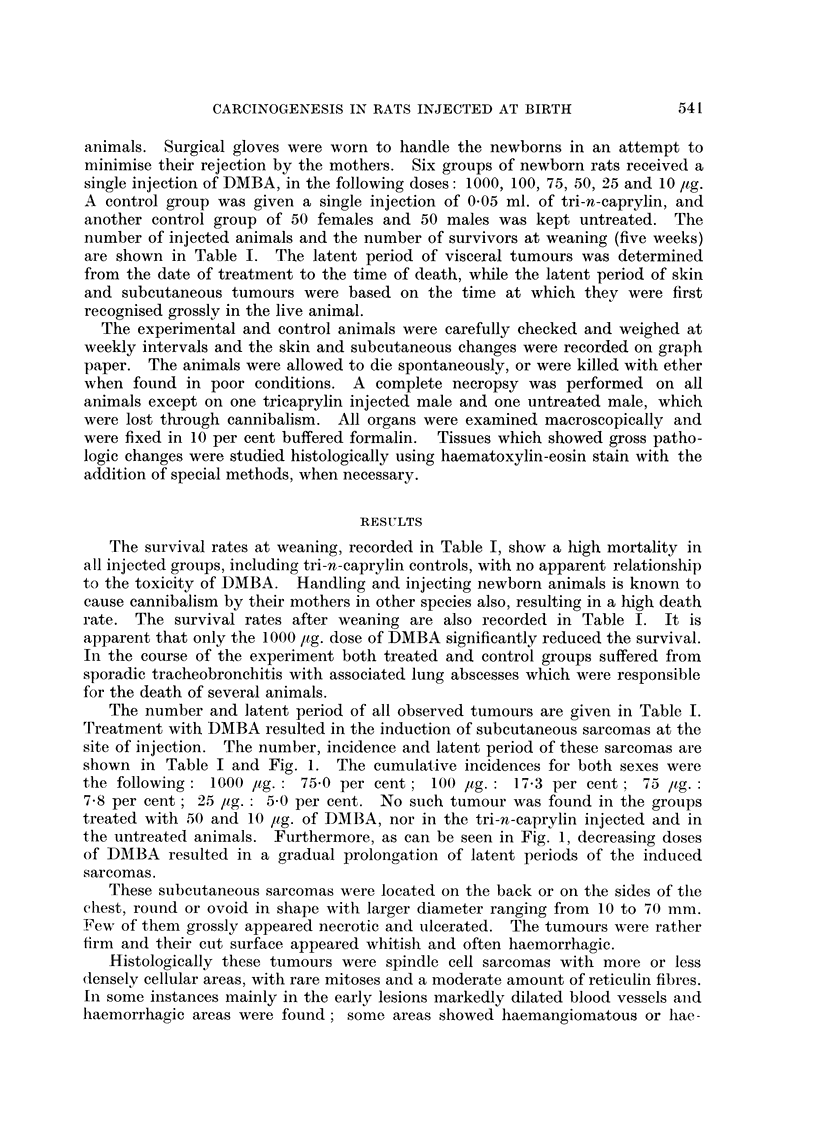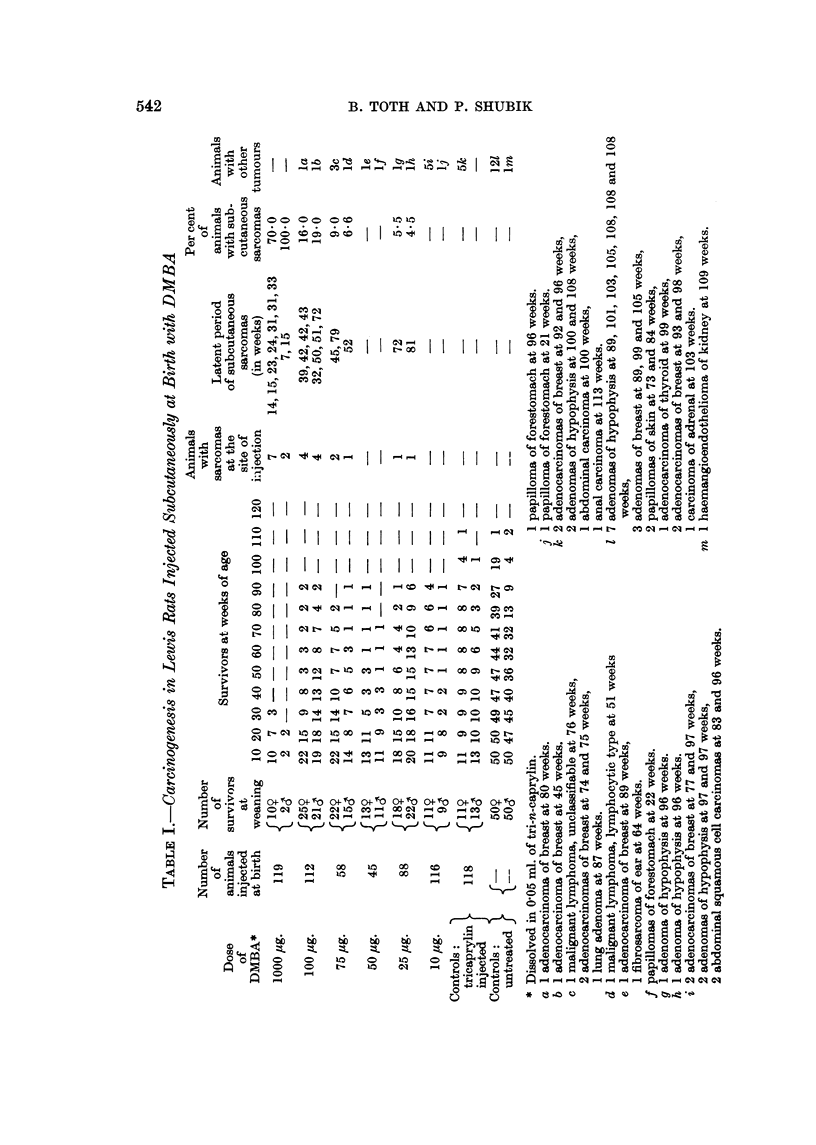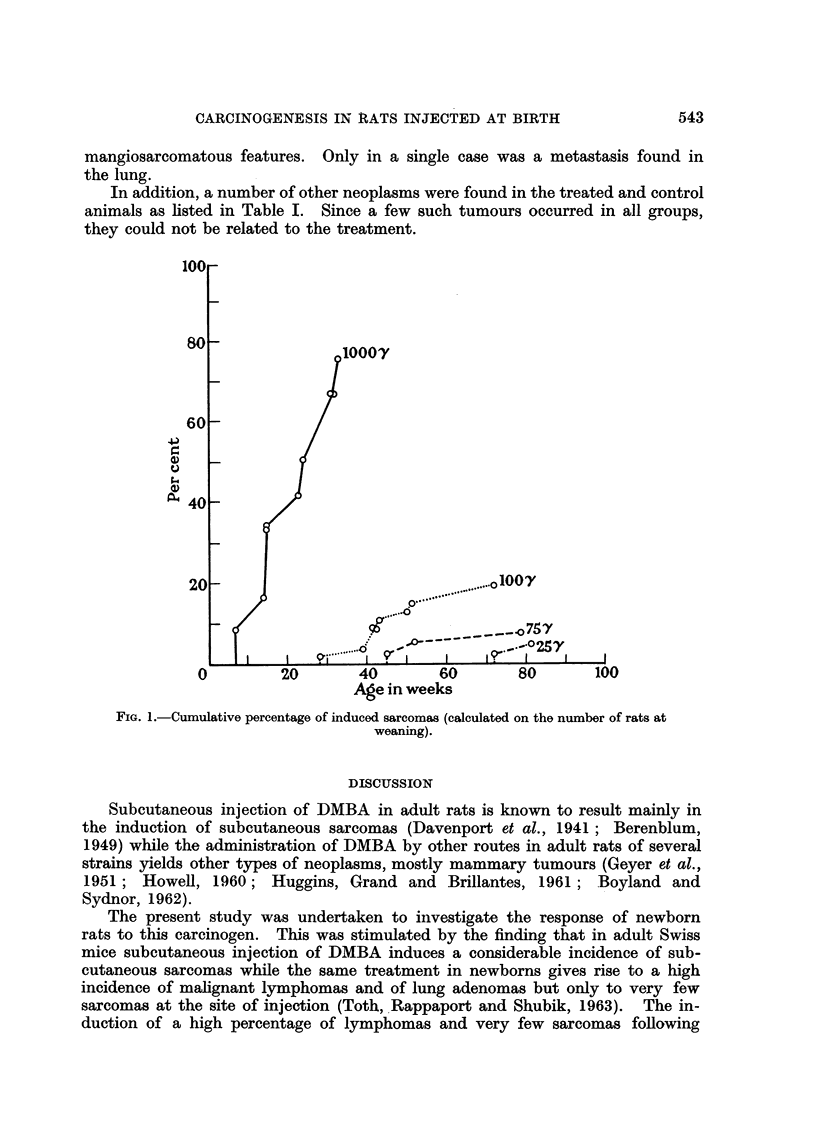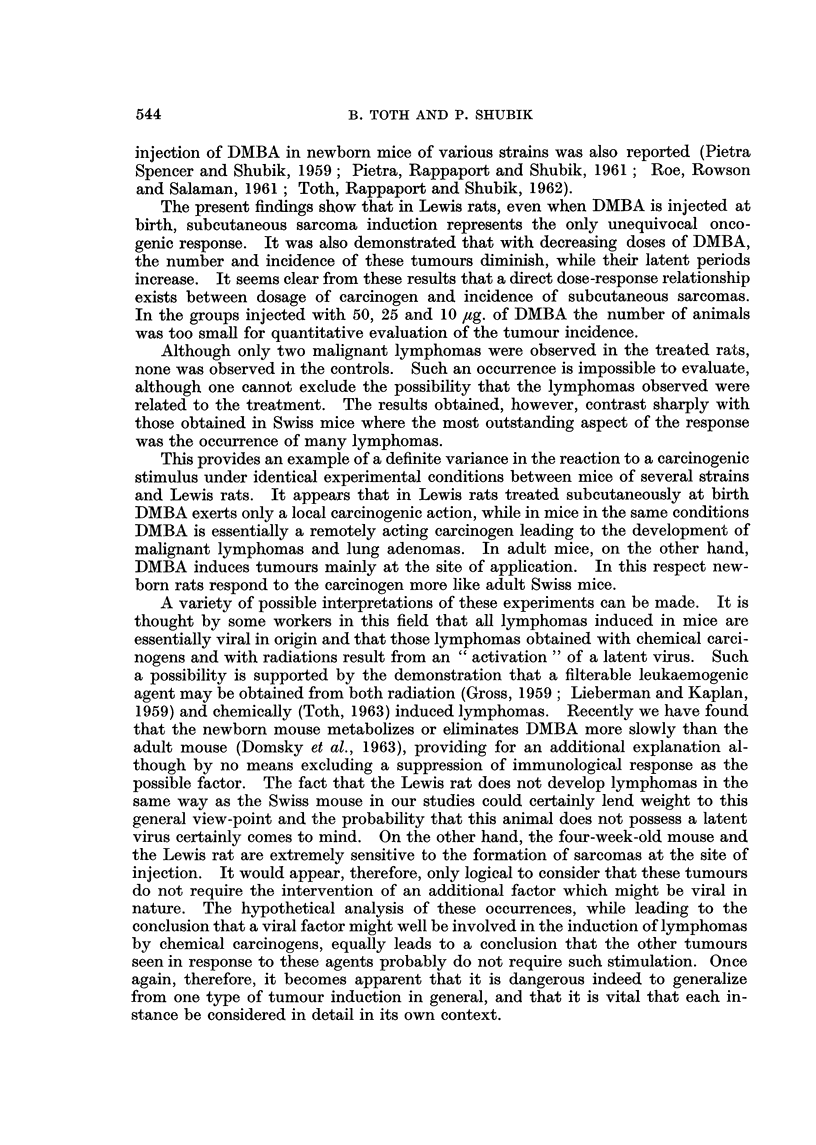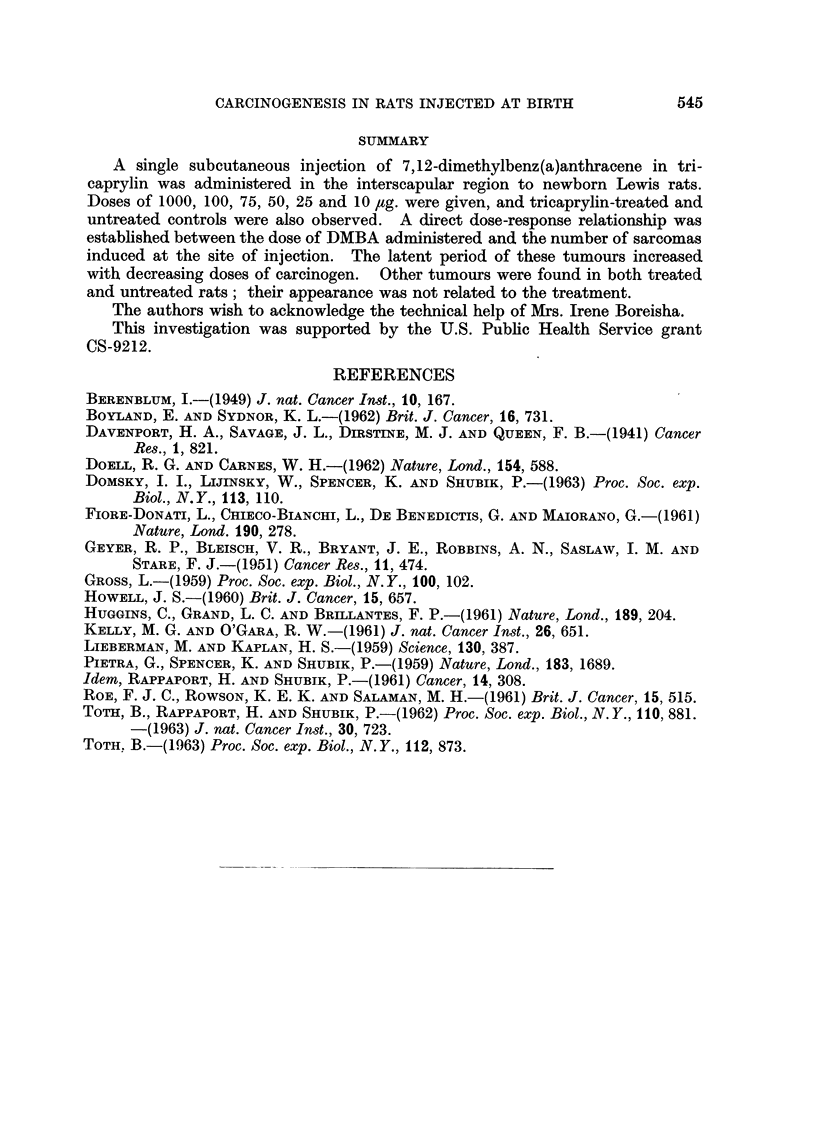# Carcinogenesis in Lewis Rats Injected at Birth with 7,12-Dimethylbenz(a)anthracene

**DOI:** 10.1038/bjc.1963.72

**Published:** 1963-09

**Authors:** B. Toth, P. Shubik


### CARCINOGENESIS IN LEWIS RATS INJECTED AT BIRTH WITH 7,12-DIMETHYLBENZ(a)ANTHRACENE

B. TOTH AND P. SHUBIK

*From the Chicago Medical School, Institute of Medical Research, Division of Oncology, Chicago 12, Illinois, U.S.A.*

Received for publication May 17, 1963

A VARIETY of strains of mice respond to the administration of chemical carcinogens at birth with the development of a high incidence of malignant lymphomas and of certain other tumours (Pietra, Spencer and Shubik, 1959; Pietra, Rappaport and Shubik, 1961; Kelly and O'Gara, 1961; Fiore-Donati *et al.*, 1961; Roe, Rowson and Salaman, 1961; Toth, Rappaport and Shubik, 1962; Doell and Carnes, 1962). The carcinogen 7,12-dimethylbenz(a)anthracene (DMBA) was found to be a very potent leukaemogenic agent for mice when injected subcutaneously at birth (Pietra, Spencer and Shubik, 1959; Pietra, Rappaport and Shubik, 1961; Roe, Rowson and Salaman, 1961; Toth, Rappaport and Shubik, 1962). Its effects at different dose levels and on mice of different age have been studied (Toth, Rappaport and Shubik, 1963).

In adult rats of various strains subcutaneous administration of DMBA was found to induce mainly sarcomas at the site of injection (Davenport *et al.*, 1941; Berenblum, 1949) while administration of this carcinogen by other routes resulted mainly in the development of mammary tumours (Geyer *et al.*, 1951; Howell, 1960; Huggins, Grand and Brillantes, 1961; Boyland and Sydnor, 1962).

The present experiment was undertaken to investigate the effect of DMBA in Lewis rats, when administered subcutaneously at birth at different dose levels.

### MATERIALS AND METHODS

Lewis inbred rats originally obtained through the courtesy of Dr. Kurt Stern, Department of Pathology, University of Illinois, Chicago, and since 1961 bred in our laboratory by brother-to-sister mating, were used. Each litter was housed with its mother until it was weaned, then separated according to sex. All rats were housed in plastic cages with granular cellulose bedding in groups of five, and were given Rockland diet in pellets and tap water *ad libitum*.

The carcinogen used was 7,12-dimethylbenz(*a*)anthracene (DMBA) (Eastman Organic Chemicals), purified by chromatography on magnesia/Celite. It was dissolved in tri-*n*-caprylin (trioctanoin) (Eastman Organic Chemicals), purified by vacuum distillation, at different concentrations, such that the desired dose of DMBA was always contained in 0·05 ml. of tri-*n*-caprylin. The animals were injected subcutaneously in the interscapular area with a tuberculin syringe, using a 30 gauge needle. The injections were made less than 24 hours after birth of the animals. Surgical gloves were worn to handle the newborns in an attempt to minimise their rejection by the mothers. Six groups of newborn rats received a single injection of DMBA, in the following doses: 1000, 100, 75, 50, 25 and 10 $\mu$g. A control group was given a single injection of 0·05 ml. of tri-*n*-caprylin, and another control group of 50 females and 50 males was kept untreated. The number of injected animals and the number of survivors at weaning (five weeks) are shown in Table I. The latent period of visceral tumours was determined from the date of treatment to the time of death, while the latent period of skin and subcutaneous tumours were based on the time at which they were first recognised grossly in the live animal.

The experimental and control animals were carefully checked and weighed at weekly intervals and the skin and subcutaneous changes were recorded on graph paper. The animals were allowed to die spontaneously, or were killed with ether when found in poor conditions. A complete necropsy was performed on all animals except on one tricaprylin injected male and one untreated male, which were lost through cannibalism. All organs were examined macroscopically and were fixed in 10 per cent buffered formalin. Tissues which showed gross pathologic changes were studied histologically using haematoxylin-eosin stain with the addition of special methods, when necessary.

### RESULTS

The survival rates at weaning, recorded in Table I, show a high mortality in all injected groups, including tri-*n*-caprylin controls, with no apparent relationship to the toxicity of DMBA. Handling and injecting newborn animals is known to cause cannibalism by their mothers in other species also, resulting in a high death rate. The survival rates after weaning are also recorded in Table I. It is apparent that only the 1000 $\mu$g. dose of DMBA significantly reduced the survival. In the course of the experiment both treated and control groups suffered from sporadic tracheobronchitis with associated lung abscesses which were responsible for the death of several animals.

The number and latent period of all observed tumours are given in Table I. Treatment with DMBA resulted in the induction of subcutaneous sarcomas at the site of injection. The number, incidence and latent period of these sarcomas are shown in Table I and Fig. 1. The cumulative incidences for both sexes were the following: 1000 $\mu$g.: 75·0 per cent; 100 $\mu$g.: 17·3 per cent; 75 $\mu$g.: 7·8 per cent; 25 $\mu$g.: 5·0 per cent. No such tumour was found in the groups treated with 50 and 10 $\mu$g. of DMBA, nor in the tri-*n*-caprylin injected and in the untreated animals. Furthermore, as can be seen in Fig. 1, decreasing doses of DMBA resulted in a gradual prolongation of latent periods of the induced sarcomas.

These subcutaneous sarcomas were located on the back or on the sides of the chest, round or ovoid in shape with larger diameter ranging from 10 to 70 mm. Few of them grossly appeared necrotic and ulcerated. The tumours were rather firm and their cut surface appeared whitish and often haemorrhagic.

Histologically these tumours were spindle cell sarcomas with more or less densely cellular areas, with rare mitoses and a moderate amount of reticulin fibres. In some instances mainly in the early lesions markedly dilated blood vessels and haemorrhagic areas were found; some areas showed haemangiomatous or hae-

TABLE I.—*Carcinogenesis in Lewis Rats Injected Subcutaneously at Birth with DMBA*

mangiosarcomatous features. Only in a single case was a metastasis found in the lung.

In addition, a number of other neoplasms were found in the treated and control animals as listed in Table I. Since a few such tumours occurred in all groups, they could not be related to the treatment.

FIG. 1.—Cumulative percentage of induced sarcomas (calculated on the number of rats at weaning).

DISCUSSION

Subcutaneous injection of DMBA in adult rats is known to result mainly in the induction of subcutaneous sarcomas (Davenport *et al.*, 1941 ; Berenblum, 1949) while the administration of DMBA by other routes in adult rats of several strains yields other types of neoplasms, mostly mammary tumours (Geyer *et al.*, 1951 ; Howell, 1960 ; Huggins, Grand and Brillantes, 1961 ; Boyland and Sydnor, 1962).

The present study was undertaken to investigate the response of newborn rats to this carcinogen. This was stimulated by the finding that in adult Swiss mice subcutaneous injection of DMBA induces a considerable incidence of subcutaneous sarcomas while the same treatment in newborns gives rise to a high incidence of malignant lymphomas and of lung adenomas but only to very few sarcomas at the site of injection (Toth, Rappaport and Shubik, 1963). The induction of a high percentage of lymphomas and very few sarcomas following injection of DMBA in newborn mice of various strains was also reported (Pietra Spencer and Shubik, 1959 ; Pietra, Rappaport and Shubik, 1961 ; Roe, Rowson and Salaman, 1961 ; Toth, Rappaport and Shubik, 1962).

The present findings show that in Lewis rats, even when DMBA is injected at birth, subcutaneous sarcoma induction represents the only unequivocal oncogenic response. It was also demonstrated that with decreasing doses of DMBA, the number and incidence of these tumours diminish, while their latent periods increase. It seems clear from these results that a direct dose-response relationship exists between dosage of carcinogen and incidence of subcutaneous sarcomas. In the groups injected with 50, 25 and 10 $\mu$g. of DMBA the number of animals was too small for quantitative evaluation of the tumour incidence.

Although only two malignant lymphomas were observed in the treated rats, none was observed in the controls. Such an occurrence is impossible to evaluate, although one cannot exclude the possibility that the lymphomas observed were related to the treatment. The results obtained, however, contrast sharply with those obtained in Swiss mice where the most outstanding aspect of the response was the occurrence of many lymphomas.

This provides an example of a definite variance in the reaction to a carcinogenic stimulus under identical experimental conditions between mice of several strains and Lewis rats. It appears that in Lewis rats treated subcutaneously at birth DMBA exerts only a local carcinogenic action, while in mice in the same conditions DMBA is essentially a remotely acting carcinogen leading to the development of malignant lymphomas and lung adenomas. In adult mice, on the other hand, DMBA induces tumours mainly at the site of application. In this respect newborn rats respond to the carcinogen more like adult Swiss mice.

A variety of possible interpretations of these experiments can be made. It is thought by some workers in this field that all lymphomas induced in mice are essentially viral in origin and that those lymphomas obtained with chemical carcinogens and with radiations result from an " activation " of a latent virus. Such a possibility is supported by the demonstration that a filterable leukaemogenic agent may be obtained from both radiation (Gross, 1959 ; Lieberman and Kaplan, 1959) and chemically (Toth, 1963) induced lymphomas. Recently we have found that the newborn mouse metabolizes or eliminates DMBA more slowly than the adult mouse (Domsky *et al.*, 1963), providing for an additional explanation although by no means excluding a suppression of immunological response as the possible factor. The fact that the Lewis rat does not develop lymphomas in the same way as the Swiss mouse in our studies could certainly lend weight to this general view-point and the probability that this animal does not possess a latent virus certainly comes to mind. On the other hand, the four-week-old mouse and the Lewis rat are extremely sensitive to the formation of sarcomas at the site of injection. It would appear, therefore, only logical to consider that these tumours do not require the intervention of an additional factor which might be viral in nature. The hypothetical analysis of these occurrences, while leading to the conclusion that a viral factor might well be involved in the induction of lymphomas by chemical carcinogens, equally leads to a conclusion that the other tumours seen in response to these agents probably do not require such stimulation. Once again, therefore, it becomes apparent that it is dangerous indeed to generalize from one type of tumour induction in general, and that it is vital that each instance be considered in detail in its own context.

SUMMARY

A single subcutaneous injection of 7,12-dimethylbenz(a)anthracene in tricaprylin was administered in the interscapular region to newborn Lewis rats. Doses of 1000, 100, 75, 50, 25 and 10 μg. were given, and tricaprylin-treated and untreated controls were also observed. A direct dose-response relationship was established between the dose of DMBA administered and the number of sarcomas induced at the site of injection. The latent period of these tumours increased with decreasing doses of carcinogen. Other tumours were found in both treated and untreated rats ; their appearance was not related to the treatment.

The authors wish to acknowledge the technical help of Mrs. Irene Boreisha.

This investigation was supported by the U.S. Public Health Service grant CS-9212.

### REFERENCES

Berenblum, I.—(1949) *J. nat. Cancer Inst.*, **10**, 167.

Boyland, E. and Sydnor, K. L.—(1962) *Brit. J. Cancer*, **16**, 731.

Davenport, H. A., Savage, J. L., Dirstine, M. J. and Queen, F. B.—(1941) *Cancer Res.*, **1**, 821.

Doell, R. G. and Carnes, W. H.—(1962) *Nature, Lond.*, **154**, 588.

Domsky, I. I., Lijinsky, W., Spencer, K. and Shubik, P.—(1963) *Proc. Soc. exp. Biol., N.Y.*, **113**, 110.

Fiore-Donati, L., Chieco-Bianchi, L., De Benedictis, G. and Maiorano, G.—(1961) *Nature, Lond.* **190**, 278.

Geyer, R. P., Bleisch, V. R., Bryant, J. E., Robbins, A. N., Saslaw, I. M. and Stare, F. J.—(1951) *Cancer Res.*, **11**, 474.

Gross, L.—(1959) *Proc. Soc. exp. Biol., N.Y.*, **100**, 102.

Howell, J. S.—(1960) *Brit. J. Cancer*, **15**, 657.

Huggins, C., Grand, L. C. and Brillantes, F. P.—(1961) *Nature, Lond.*, **189**, 204.

Kelly, M. G. and O'Gara, R. W.—(1961) *J. nat. Cancer Inst.*, **26**, 651.

Lieberman, M. and Kaplan, H. S.—(1959) *Science*, **130**, 387.

Pietra, G., Spencer, K. and Shubik, P.—(1959) *Nature, Lond.*, **183**, 1689.

*Idem*, Rappaport, H. and Shubik, P.—(1961) *Cancer*, **14**, 308.

Roe, F. J. C., Rowson, K. E. K. and Salaman, M. H.—(1961) *Brit. J. Cancer*, **15**, 515.

Toth, B., Rappaport, H. and Shubik, P.—(1962) *Proc. Soc. exp. Biol., N.Y.*, **110**, 881.
—(1963) *J. nat. Cancer Inst.*, **30**, 723.

Toth, B.—(1963) *Proc. Soc. exp. Biol., N.Y.*, **112**, 873.